# A Case of Puerperal Eclampsia

**Published:** 1887-12

**Authors:** 


### A CASE OF PUERPERAL ECLAMPSIA.

### INDUCTION OF LABOR.—RECOVERY.

CASE IV.

H—— G——, white, single, aged 25 years, and of Swedish descent was admitted to the obstetrical ward of the hospital, October 17th, 1887.

Her family history is good; there are no neurotic tendencies. The patient had been troubled with dysmenorrhœa, probably obstructive, for 10 years previous to the date of her last menstruation, on February 10th, 1887.

During the greater part of her gestation she was a sufferer from headache, backache, anorexia, neuralgia and general weakness, which at times necessitated her keeping her bed for several weeks.

About September 1st, she noticed that her face, and, at times, her hands, were puffed after lying down. During September and October she suffered from severe morning vomiting and coronal headache; and experienced several "spells," one of which was attended with unconsciousness, which was relieved only by venesection.

October 24th. Urine: yellow, acid, sp. gr. 1003, trace of albumen. Amount passed in 24 hours, 50 ozs., containing 165 grains of solids.

Ordered: Hydrarg. bichlor, gr. $\frac{1}{64}$, Potass. Iod. grs. X. 3 times daily, with Tr. Ferri chloridi M xv and Sp. Frumenti, ʒss, after meals.

November 3d. Urine: yellow, acid, sp. gr. 1010, no albumen, amount for 24 hours, 80 ozs., containing 880 grains of solids.

After her admission into the hospital she had several attacks of syncope, of which she afterwards had no recollection.

November 15th, at 10 P. M., she had a typical eclamptic seizure. She was unconscious for two hours.

February 16th. She was very nervous and apprehensive. Efforts at vomiting had been so constant and so severe that she was quite exhausted—merely touching the cervix would excite nausea. It was then decided to induce labor, chloroform was administered and with fingers os uteri was forcibly dilated and the membranes ruptured. A vertex presentation in the third (O.D.P.) position was diagnosed.

Miller's forceps were applied, and the child delivered in 25 minutes. Good retraction was secured by the administration of ergotin, gr. iii, hypodermatically.

The perinæum was lacerated down to sphincter ani, and coaptated with one internal continuous catgut and five external silk sutures, which were removed on the 8th day when union was perfect.

The child was a male, vigorous and weighing seven pounds; it was completely resuscitated in 15 minutes. Four convulsions occurred during the puerperal period, two of which were controlled by morph. sulph. gr. ¼, administered hypodermatically. Two were attended with unconsciousness, when morphia sulph. gr. ½ was administered and followed by fl. ext. pilocarpine ʒss. which produced free diaphoresis with subsidence of all unfavorable symptoms. November 24th. Urine:—Deep yellow, acid, sp. gr. 1025, trace of albumen. 32 ounces passed in 24 hours, containing 880 grains of solids. The hydrarg, bichlor. and the potass. iod. were discontinued, and potass. acet., grs. xxx, with potass. et sod. tartras xi. to be given every four hours was ordered. The usual strict antiseptic precautions now in practice in the obstetric ward were observed throughout the management of the case. The temperature at no time exceeded 99.8° F. Her bowels were moved daily by a glycerine and water enema, or by some mild laxative. The severe headaches were relieved by 15 grain doses of antipyrin. After the last convulsion the patient improved rapidly. Her supply of milk was scanty, and as she could not keep the child, its secretion was suppressed by the application of ungt. belladonnæ and uniform compression to the breasts.

December 12.—She has good appetite, sleeps well, is gaining flesh rapidly and feels perfectly well.

